# Inhibition of Ubiquitin Specific Protease 1 Sensitizes Colorectal Cancer Cells to DNA-Damaging Chemotherapeutics

**DOI:** 10.3389/fonc.2019.01406

**Published:** 2019-12-18

**Authors:** Xin Xu, Shaoyan Li, Ximao Cui, Kunkun Han, Jun Wang, Xiaodan Hou, Long Cui, Songbing He, Jiecheng Xiao, Yili Yang

**Affiliations:** ^1^Center for Systems Medicine, Suzhou Institute of Systems Medicine, Chinese Academy of Medical Sciences, Suzhou, China; ^2^School of Pharmacy, China Pharmaceutical University, Nanjing, China; ^3^Department of Colorectal Surgery, Xinhua Hospital, Shanghai Jiaotong University School of Medicine, Shanghai, China; ^4^Shanghai Colorectal Cancer Research Center, Shanghai, China; ^5^The Asclepius Technology Company Group and Asclepius Cancer Research Center, Suzhou, China; ^6^The First Affiliated Hospital of Soochow University, Suzhou, China; ^7^State Key Laboratory of Innovative Natural Medicine and TCM Injections, Qingfeng Pharmaceutical, Ganzhou, China

**Keywords:** deubiquitinating enzymes, ubiquitin specific protease 1, USP1, colorectal cancer, chemotherapy

## Abstract

Mutations and altered expression of deubiquitinating enzymes (DUBs) have been found associated with many human diseases including cancers. In this study, Ubiquitin specific protease 1 (USP1) expression was found significantly increased in some colorectal cancers (CRC). The elevated USP1 level was associated with short overall survival of patients and with advanced stages of cancers. In cultured CRC cells, knockdown of USP1 induced growth arrest at G_2_/M of cell cycle and reduced the expression of anti-apoptotic proteins Bcl-2 and Mcl-1. Its knockdown also led to reduction of DNA-repair related substrates FANCD2 and ID1. Further investigations found that small molecular inhibitor of USP1 ML323 sensitized CRC cells to DNA-targeting chemotherapeutics, including doxorubicin, TOPI/II inhibitors, and PARP inhibitor, but not to 5-Fu. These results indicate that USP1 plays a critical in colorectal cancer cell survival and is a promising target for anti-colorectal cancer chemotherapy. Targeting USP1 may represent an effective strategy to regulate the DNA-repairing system.

## Introduction

Ubiquitination, the addition of ubiquitin through isopeptide bond to target proteins, usually requires the sequential actions of ubiquitin-activating enzyme (E1), ubiquitin-conjugating enzyme (E2), and ubiquitin ligase (E3). It controls the level, location, and activity of many proteins and participates in most, if not all, cellular processes, including signal transduction, cell cycle, growth, and apoptosis (1). It has been shown that dysfunction of the ubiquitination and proteasomal degradation system, particularly the degradation of tumor suppressors and the elevation of oncoproteins, play a critical role in cancer initiation and progression (2). Of note, protein ubiquitination can be reversed by a family of proteases named deubiquitinating enzymes (DUBs), among which, the largest subfamily is the ubiquitin-specific proteases (USPs) (3). In addition to their protease region, many USPs also contain various functional domains such as ubiquitin-binding motif and Zinc finger, indicating that each USP may interact with different proteins and participate in unique cellular processes. It has been shown that mutations and altered expression of DUBs are closely associated with various human cancers (4). Since most of these DUBs are cysteine proteases and reactive to various electrophiles, they have been the preferred targets for developing novel chemotherapeutics. Therefore, it is highly desirable to characterize and understand DUBs' criticality for each cancer in precision cancer treatment.

Ubiquitin specific protease 1 (USP1) is a 785 amino-acid deubiquitinating enzyme that contains the characteristic His and Cys domains which are highly conserved in all members of the ubiquitin-specific processing (UBP) family of proteases (5). It has been shown that the ubiquitination status of Fanconi anemia protein FANCD2, a critical component of DNA damage repair, is regulated by USP1 (6). While DNA-dependent mono-ubiquitination of FANCD2 facilitates DNA repair, it is deubiquitinated by USP1 to extinguish the timely DNA-repairing response. It is evident that USP1 also played an important role in DNA translesion synthesis through regulating the mono-ubiquitination of PCNA (7, 8), and promoted homologous recombination to repair DNA double strand breaks (9). The importance of these findings was further demonstrated by the observation that USP1 deficient mice were highly sensitive to DNA damage (10). Interestingly, the expression of USP1 was significantly increased in a number of cancers (11, 12), and blocking USP1 inhibited DNA repair, induced apoptosis in multiple myeloma cells (13), and sensitized lung cancer cells to cisplatin (14, 15). These results indicated that USP1 is a promising target for chemotherapy of at least some cancers.

Colorectal cancer (CRC) is among the most common malignancies in both man and woman worldwide (16). Despite significant progress in understanding the underlying molecular changes and advances in the treatment, the 5-year survival rate of patients with advanced CRC remains significantly below 50% (17). Finding new targets and approaches for its treatment is sorely needed to improve the prognosis of these patients. In this study, we examined the expression of USP1 in CRC and investigated the effects of altered USP1 expression on CRC cells and assessed the role of knockdown and inhibition of USP1 on the cytotoxic action of chemotherapeutics. These results indicated that USP1 was over-expressed at least in some primary CRC and played an important role in CRC cell growth, and survival, and targeting USP1 was an effective anti-CRC strategy that deserved further exploration.

## Materials and Methods

### Cells, Tissues, and Chemicals

CRC cell lines HCT116, HT-29, SW948, SW620, and SW480 were purchased from American Type Culture Collection (Manassas, VA). They were maintained in Dulbecco's high glucose modified Eagle's medium (DMEM) supplemented with 10% fetal bovine serum, 100 μg/ml of penicillin, and 100 units/ml of streptomycin. The primary CRC tissues and paracancerous normal tissues were collected from CRC patients enrolled in the Department of Colorectal Surgery, Xinhua Hospital, Shanghai Jiaotong University. The collection and use of human tissues for this study were approved by the Institutional Review Board and with informed consent. Doxorubicin was purchased from Sigma-Aldrich (St. Louis, MO). PARPi (Olaparib), CAM (Camptothecin), Eto (Etoposide), AMO (Amonafide), and 5-FU were purchased from Selleck Chemicals (Houston, USA).

### Plasmids Construction and Gene Transfection

The human USP1 gene was generated and cloned into pcDNA3.1 vector with a Flag tag as previously described (18). Single Cysteine (C) to Serine (S) mutant USP1-C90S was constructed by using a PCR-based site-directed mutagenesis kit (19). The primers for generating USP1 and USP1-C90S plasmids were as follows: USP1, Forward 5′-CGGGATCCATGCCTGGTGTCATACCTAGT-3′, Reverse 5′-CCGCTCGAGCTATAATTTCTTATAAAATAG-3′; USP1-C90S, Forward 5′-ATCTCGGCAATACTTCCTATCTTAATAGTAT-3′, Reverse 5′-ATACTATTAAGATAGGAAGTATTGCCGAGAT-3′. The siRNA against USP1 and the negative control (siNC) were synthesized by Guangzhou Ribobio (Guangzhou, China) as reported previously (13).

Plasmids or siRNAs were transiently transfected into HCT116 or SW620 cells by Lipofectamine 2000 (Invitrogen) according to the manufacturer's instruction.

### Preparation of shRNA Lentivirus

The lentivirus-delivered shRNAs against USP1 (shUSP1) and the negative control (shNC) were purchased from Shanghai GeneChem Co., Ltd. (Shanghai, China). The target sequence of shUSP1 was 5′-CCAGTGACCAAACAGGCATTA-3′. The viral particles were prepared with a standard protocol as manufacturer's instructions.

### Immunoblotting

Cell lysates were prepared for immunoblotting as described (20–22). The antibodies against USP1, CyclinD1, and α-Tublin were purchased from Santa Cruz Biotechnology (Santa Cruz, CA). The antibodies against CDK4, CDK6, p-p53, p21, and cleaved-PARP were purchased from Cell Signaling Technology (Danvers, MA). Anti-Flag antibody was purchased from Medical & Biological Laboratories (Tokyo, Japan). Anti-GAPDH antibody was purchased from Abgent (Suzhou, China). Horseradish peroxidase conjugated anti-mouse IgG and anti-rabbit IgG antibodies were purchased from Beyotime Biotechnology (Nantong, China).

### Quantitative Real-Time Polymerase Chain Reaction

Total RNA was extracted using RNAiso Plus (Takara Bio Group, Japan). cDNA was synthesized from the total RNA using the PrimeScriptTM RT reagent Kit (Takara Bio Group, Japan) as described previously (23). To determine the mRNA levels of USP1, CyclinA1, CyclinD1, CyclinE1, Bcl2, and Mcl1, qRT-PCR was performed using SYBR Green qPCR Master Mix (Clontech, USA) with Roche LightCycler® 480II real-time PCR system (Roche, Basel, Switzerland). The primers used were as follows: USP1, forward 5′-ATACTGAAGCTGAACGAAGTC-3′ and reverse 5′-GATCTTGGAAAGTCCACCAC-3′; CyclinA1, forward 5′-ACTGCTGCTATGCTGTTA-3′ and reverse 5′-TGGTGTAGGTATCATCTGTAAT-3′; CyclinD1, forward 5′-CTCTAAGATGAAGGAGACCAT-3′ and reverse 5′-TTGGAGAGGAAGTGTTCAA-3′; CyclinE1, forward 5′-CAGCCAAACTTGAGGAAAT-3′ and reverse 5′- TCAGCCAGGACACAATAG-3′; Bcl-2, forward 5′-ATGACTGAGTACCTGAACC-3′ and reverse 5′-AGACAGCCAGGAGAAATC-3′; Mcl1, forward 5′-GAACCATTAGCAGAAAGTATCA-3′ and reverse 5′-ATCCCAGCCTCTTTGTTTA-3′; GAPDH, forward 5′-GGTATCGTGGAAGGACTCATGAC-3′ and reverse 5′-ATGCCAGTGAGCTTCCCGTTCAG-3′; β-actin, forward 5′-CACCCAGCACAATGAAGATC-3′ and reverse 5′-CATACTCCTGCTTGCTGATC-3′.

### Cell Cycle Analysis

HCT116 cells were infected with shNC or shUSP1 for 36 h before being harvested for cell cycle analysis as described previously (24). Briefly, cells were fixed with 70% cold ethanol overnight and washed once with phosphate buffered saline (PBS), followed by resuspending in 100 μl PBS containing 100 μg/ml RnaseA (Beyotime Biotechnology, Nantong, China). Cells were then washed once with cold PBS and incubated with nuclear staining propidium iodide (PI) for 5 min at a final concentration of 50 μg/ml. The cell cycle was analyzed on a flow cytometer (Thermo Fisher, USA).

### Cell Growth and Viability

CRC cells were transfected with siRNA or plasmids for 24 h. They were then treated with DOX, Olaparib, CAM, Eto, AMO, or 5-FU for 24 h at indicated concentrations in the presence or absence of 50 μM ML323. The viable cells were evaluated by Cell Counting Kit-8 (CCK-8) staining according to the manufacturer's instructions (Biotool, Houston).

### Immunohistochemistry Analysis

CRC tissues and paracancerous tissues were fixed in 10% formalin before being embedded in paraffin. Then, the tissues were cut into 6-micron thickness with a microtome, and the slides were deparaffinized and rehydrated before antigen retrieval. All slides were then subjected to blocking in 10% normal horse serum for 10 min, followed by incubating with the primary antibody USP1 (1:100; Santa Cruz Biotechnology, US) overnight at 4°C. Next, a biotin-conjugated secondary antibody diluted with Tris-based buffer (TBS) containing 10% serum and 1% BSA was applied to incubate for 3 min at room temperature. The slides were rinsed with cold TBS and incubated with streptavidin-peroxidase for 10 min before being stained with 3,3′-Diaminobenzidine (Invitrogen, USA). The slides were finally stained with 1% hematoxylin and sealed with coverslip before being mounted for microscopy analysis. USP1 staining was graded according to the following criteria: 0 (no staining), 1 (mild staining), or 3 (strongly positive). Two histopathologists blinded to the clinical data were assigned to review and score the slides.

### Survival Curve Analysis

To evaluate the survival time of CRC patients in association with USP1, 169 patients were divided into two groups according to the scores of USP1 expression (USP1 low: 0, 1; USP1 high: 2, 3). The survival curve was calculated according to the Kaplan-Meier estimates.

### Statistical Analysis

Statistical analysis and graphical representation were performed using Prism version 6.0 (GraphPad, La Jolla, CA, USA). The chi-square test was used to evaluate the association between USP1 expression in CRC samples and the clinic pathological parameters, and the Kaplan-Meier method and logrank test were used for survival analysis. The student's *t* test was used for comparisons of two groups in the studies. All statistical tests were two-sided, and a *p* < 0.05 was considered statistically significant.

## Results

### Ubiquitin Specific Protease 1 Is Highly Expressed in Some Colorectal Cancers

**Figure 1:**
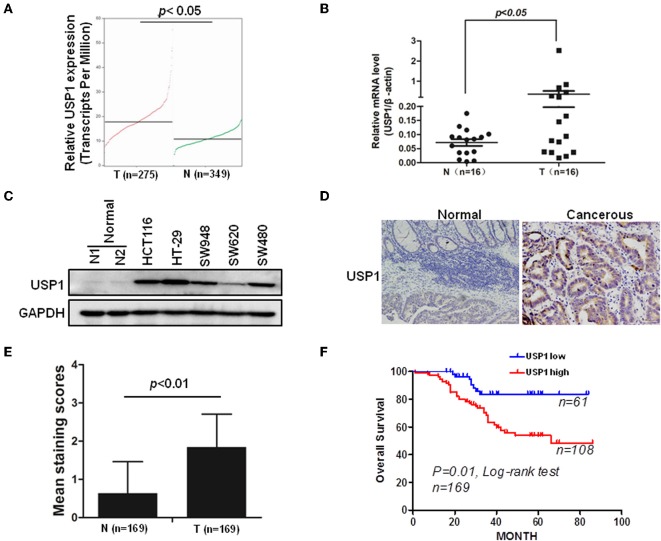
Ubiquitin specific protease (USP1) expression is increased in colorectal cancer and predicts a poor prognosis of patients with colorectal cancer. **(A)** The expression level of USP1 in colorectal adenocarcinoma (COAD) was analyzed by Gene Expression Profiling Interactive Analysis (GEPIA), matched with public cancer database The Cancer Genome Atlas (TCGA) and The Genotype-Tissue Expression (GTEx) (http://gepia.cancer-pku.cn). N, normal; T, tumor. **(B)** Sixteen pairs of paracancerous and tumor tissues from colorectal cancer were collected and examined for USP1 expression by qRT-PCR. β-actin was used as an internal control. **(C)** Five colorectal cancer cell lines and two paracancerous tissues were lysed for immunoblotting against USP1. Glyceraldehyde-3-phosphate dehydrogenase (GAPDH) was used as an internal control. **(D)** Representative immunohistochemical fields of human colorectal cancer tissues stained with an anti-USP1 antibody. **(E)** Statistical analysis of human colorectal cancer tissue array (*n* = 169) stained with an anti-USP1 antibody. Immunostaining scores (mean ± SD) for USP1 in paracancerous (N) and cancerous (T) tissues were summarized. **(F)** The Kaplan–Meier survival curves of colorectal cancer patients. There was a significant difference between these with tumors expressing high and low levels of USP1.

In an effort to find deubiquitinating enzymes that affected the growth of CRC cells, we screened a shRNA library in our previous study and found that USP1 knockdown had a significant effect (3). The expression level of USP1 was firstly analyzed by Gene Expression Profiling Interactive Analysis (GEPIA), and the results showed that USP1 expression was significantly higher in colon adenocarcinoma compared with that of controls (*p* < 0.05) (Figure 1A). We then examined USP1 expression in primary CRC tissues and cell lines by qRT-PCR and immunoblotting. As shown in Figures 1B,C, the level of USP1 was significantly elevated in tumor tissues and in most tumor cell lines examined. CRC tissue arrays were then utilized to examine USP1 expression and survival of patients with CRC. Immunohistochemical staining revealed that CRCs expressed significantly increased level of USP1 (Figures 1D,E). In this cohort (Table 1), the CRC patients with high level of USP1 had a significant shorter survival compared with those with low USP1 expression (Figure 1F). Noteworthily, CRCs at advanced stage (IV) showed elevated level of USP1, although it was statistically not significant due to the limited numbers of patients with stage IV cancers in the cohort (Table 2).

**Table 1:** Case Information.

**Clinical parameters**		**Case (%)**
Gender	Male	91 (53.8)
	Female	78 (46.2)
Age	≤60	79 (46.7)
	>60	90 (53.3)
Stage	I	13 (7.7)
	II	66 (39.1)
	III	71 (42.0)
	IV	19 (11.2)
T	1	3 (1.8)
	2	15 (8.9)
	3	57 (33.7)
	4	94 (55.6)
N	0	85 (50.3)
	1	55 (32.5)
	2	29 (17.2)
M	0	148 (87.6)
	1	21 (12.4)
Pathology	I	31(18.3)
	II	125 (74.0)
	III	13 (7.7)

*Stage I, II, III, IV: according to NCCN; T (1, 2, 3, 4): tumor infiltration, 1 (mucous), 2 (muscular), 3 (serous), 4 (neighboring organ); M: tumor metastasis, 0 (none), 1 (yes); N: metastasized lymph node, 0 (none), 1 (local), 2 (distant); Pathology (I, II, III): high, medium, low differentiation*.

**Table 2:** IHC expression of USP1.

**Tissues**	**Cases(N)**	**Score of USP1 expression**				***P***
		0	1	2	3	
T	169	8	53	65	43	<0.0001
N	169	98	37	32	2	
Stage						
I	13	0	10	1	2	
II	66	3	23	24	16	
III	71	4	16	34	17	
IV	19	1	4	6	8	=0.007

*T: tumor; N: normal; Stage I, II, III, IV: according to NCCN*.

### USP1 Regulates Colorectal Cancer Cell Growth

**Figure 2:**
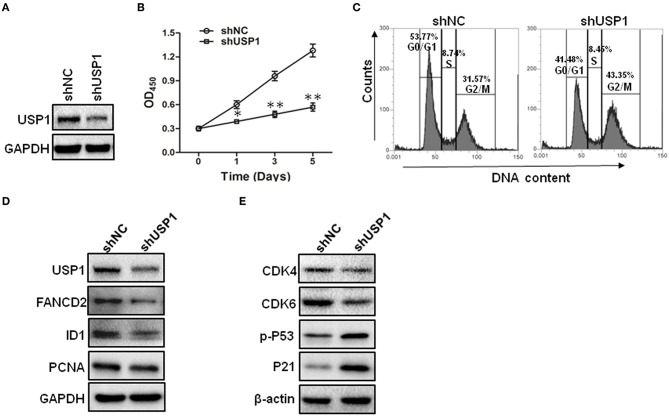
Knockdown of ubiquitin specific protease 1 (USP1) induces growth arrest in colorectal cancer cells. **(A)** HCT116 cells were stably infected with shNC or shUSP1-expressing lentivirus and prepared for immunoblotting against USP1. GAPDH was used as a loading control. **(B)** HCT116 cells were stably infected with shNC or shUSP1-expressing lentivirus, and examined with CCK-8 staining at day 0, 1, 3, or 5. **(C)** HCT116 cells were stably infected with shNC or shUSP1-expressing lentivirus and subjected to cell cycle by Propidium Iodide (PI) staining and flow cytometer analysis. **(D,E)** HCT116 cells stably infected with shNC or shUSP1-expressing lentivirus were prepared for immunoblotting against CDK4, CDK6, p-P53, P21, USP1, FANCD2, ID1, and PCNA. GAPDH and β-actin were used as a loading control. ^*^p < 0.05; ^**^p < 0.01.

The increased expression of USP1 in CRC tumors and cell lines prompted us to further investigate the role of USP1 in CRC cells. HCT116 cells were infected with lentivirus expressing shRNA targeting USP1 or control shRNA. As shown in Figures 2A,B, shUSP1 reduced the level of USP1 effectively and induced a time-dependent growth inhibition. Cell cycle analysis revealed that these cells were arrested mainly in G2/M phase (Figure 2C). Meanwhile, the expression of shUSP1 led to a reduction of FANCD2 and ID1, two of the known substrates of USP1 (Figure 2D), whereas the other reported substrate PCNA did not change significantly in the cells. As activation of p53 induces G_2_/M cell cycle block and HCT116 cells contain wild type p53 gene, we examined phosphorylated p53 and its target gene product p21, a cyclin-dependent kinase inhibitor. As shown in Figure 2E, shUSP1 markedly increased the levels of phosphorylated p53 and p21, which were likely responsible for the G_2_/M cell cycle block.

### USP1 and Apoptosis of Colorectal Cancer Cells

**Figure 3:**
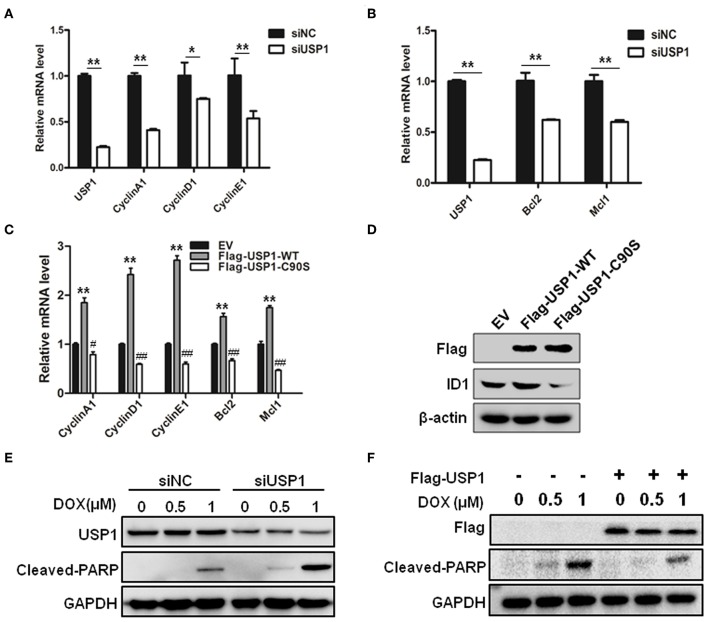
Ubiquitin specific protease 1 (USP1) regulated the expression of cell cycle and anti-apoptosis proteins. **(A)** Knockdown of USP1 decreased the expression of cyclins. siNC and siUSP1 were transfected into HCT116 cells for 24 h, followed by qRT-PCR against USP1, CyclinA1, CyclinD1, and CyclinE1. β-actin was used as an internal control. **(B)** Knockdown of USP1 reduced the expression of Bcl-2 and Mcl-1. HCT116 cells transfected with siNC or siUSP1 for 24 h were prepared for qRT-PCR against USP1, Bcl2, and Mcl1. β-actin was used as an internal control. **(C,D)** Expression of wild-type but not mutated USP1 upregulated cyclins. Empty vector (EV), Flag-USP1-WT or Flag-USP1-C90S plasmids were transfected into HCT116 cells for 48 h, followed by qRT-PCR against CyclinA1, CyclinD1, CyclinE1, Bcl2, and Mcl1 **(C)**, and immunoblotting against Flag and ID1. **(D)** β-actin was used as an internal control. **(E)** siNC and siUSP1 were transfected into HCT116 cells for 24 h. After treatment with doxorubicin (DOX) for 24 h, the cells were prepared for analyzing the expression of USP1, cleaved-poly(ADP-ribose) polymerase (PARP) and GAPDH by immunoblotting. **(F)** HCT116 cells were transfected with empty vector or Flag-USP1 for 24 h. After treatment with DOX for 24 h, the cells were analyzed by immunoblotting against Flag, cleaved-PARP and GAPDH. ^*^p < 0.05; ^**^p < 0.01; ^#^*p* < 0.05; ^##^*p* < 0.01.

As shown above, when CRC cells were stably infected with shUSP1, CRC cells with USP1 knockdown had a slow growth rate. Use was also made of siRNA targeting USP1 to evaluate the roles of USP1 in CRC cells. When CRC cells were transiently transfected with siUSP1, siUSP1 significantly downregulated the levels of Cyclin A1, D1, and E1 (Figure 3A). Interestingly, it also reduced the expression of anti-apoptosis proteins Bcl-2 and Mcl1 (Figure 3B). All the above proteins are related to cell growth and survival signals. Furthermore, while enforced expression of wild-type USP1 in HCT116 cells increased the mRNAs of Cyclin A1, D1, E1 as well as Bcl-2 and Mcl1, expression of inactive USP1 (USP1-C90S) did not affect the expression of these genes (Figure 3C). At the same time, the expression of wild-type or inactive USP1 was detected, and we also found that overexpression of wild-type USP1 markedly increased ID1 expression, one reported substrate of USP1, but the inactive USP1 did not (Figure 3D). Thus, the catalytic activity of USP1 is required for its influence on gene expression.

We then examined whether USP1 affected the drug sensitivity of doxorubicin in CRC cells. After transfection with siUSP1, HCT116 cells were treated with anti-cancer drug doxorubicin. As shown in Figure 3E, doxorubicin-induced PARP cleavage, a hallmark of cell apoptosis, was significantly increased in USP1 knockdown cells. Moreover, when USP1 was ectopically over-expressed, the cleavages of PARP induced by doxorubicin were markedly attenuated (Figure 3F). These data indicated that the inhibition of USP1 may sensitize CRC cells to apoptosis.

### USP1 Inhibitor Sensitizes Colorectal Cancer Cells to DNA-Targeting Chemotherapeutics

**Figure 4:**
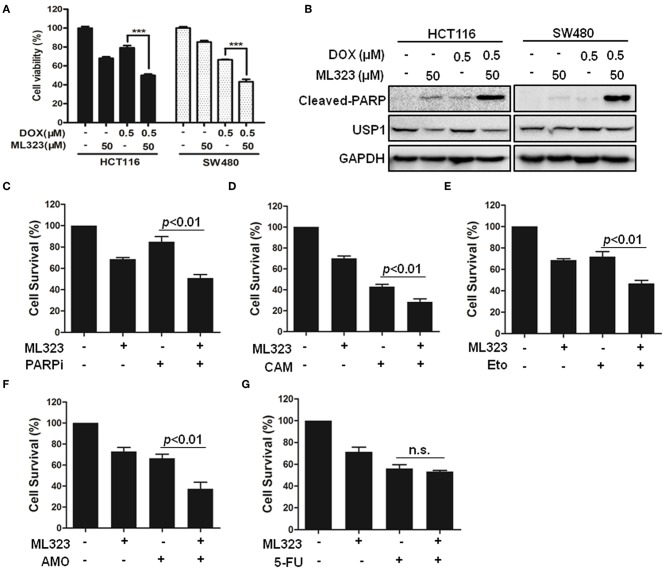
Ubiquitin specific protease 1 (USP1) inhibitor sensitized colorectal cancer cells to DNA-targeting agents doxorubicin, PARP inhibitor, etoposide, TOPI, and TOPII inhibitor in colorectal cancer cells. **(A,B)** HCT116 and SW480 cells were treated with doxorubicin (DOX) for 24 h in the presence or absence of USP1 inhibitor ML323, and then evaluated with CCK-8 staining **(A)** and immunoblotting against cleaved-PARP, USP1, and GAPDH **(B)**. **(C–G)** CCK-8 staining was used to evaluate HCT116 cells treated with 20 μM of PARP inhibitor (Olaparib) **(C)**, 10 μM of Camptothecin (CAM) **(D)**, 10 μM of etoposide **(E)**, 10 μM of Amonafide (AMO) **(F)**, 50 μg/ml of 5-FU **(G)** for 24 h in the presence or absence of 50 μM ML323. ^***^p < 0.001, n.s. means non-sense.

Given the critical roles of DUBs in various tumor cells and their catalytic characters, a variety of small molecular inhibitors of USPs have been developed as potential anti-cancer agents (4). It has been shown that ML323 was a more specific USP1 inhibitor than the previously reported inhibitors pimozide and GW7647, and it enhanced cisplatin cytotoxicity in osteosarcoma cells and non-small lung cancer cells (8). ML323 was also able to inhibit DNA repair and triggered apoptosis in myeloma cells (13). We therefore examined whether ML323 affected the effects of anti-cancer agents on CRC cells. As shown in Figures 4A,B, ML323 enhanced the cytotoxic action of doxorubicin on HCT116 and SW480 cells as measured by CCK-8 and cleavages of PARP. We then used the CCK-8 assay to further evaluate the cytotoxic action of multiple chemotherapeutic drugs. PARP inhibitor (Olaparib), topoisomerase I and II inhibitors, and DNA-binding agent etoposide all killed HCT116 cells moderately at the dosages used. In the presence of 50 μM of ML323, their effects on the cells were all significantly enhanced (Figures 4C–F). Interestingly, ML323 did not significantly enhance the anti-CRC cell action of 5-FU (Figure 4G), which acted through blocking DNA synthesis. Therefore, inhibition of USP1 sensitized CRC cells to chemotherapeutics that acted on DNA directly.

## Discussion

With about 1.4 million new cases diagnosed annually, CRC is one of the most common malignancies and a leading cause of cancer death (25). Despite significant progress in understanding the underlying molecular changes and improvement in the treatment, the prognosis of patients with CRC remains far from satisfying. Furthermore, although it has had marked downward trend in the developed world (26), the incidence rate of CRC has been increasing significantly in many developing countries and regions over the last several decades (27). It appears that the increase is largely associated with population aging, environmental contamination, and changed lifestyles. Therefore, in addition to prevention through healthy lifestyle and early diagnosis by using targeted screening, finding new effective treatment is sorely needed to improve the prognosis of individuals with CRC. It is worth noting that the development of resistance to chemotherapies is often responsible for the relapse of CRC (17), further underlining the importance of identifying novel targets and approaches for their treatment. The ubiquitin-proteasome system, which plays critical roles in cancer initiation and development, has become the focus of many investigations. Ubiquitin ligase (E3), which recognizes substrates and promotes the ubiquitin transfer, is regarded as a desired target for therapeutic intervention. However, it has become evident that E3s are difficult targets for small molecular inhibitors. Despite extensive efforts, the only inhibitor undergoing clinical trials is nutlin-3, which blocks the interaction of p53 with its E3 Mdm2 (28). Due to their critical roles in many cellular processes, it has been proposed that targeting DUBs represents an effective and relative specific means to modulate the activities of the ubiquitin-proteasome system (29). The well-defined catalytic mechanisms of various DUBs also make it possible to develop a variety of apparently druggable inhibitors (30). In the present study, we found through shRNA screening that USP1 downregulation induced growth arrest and death of CRC cells. USP1 has also been found to play important roles in cancer cells of multiple myeloma (13), leukemia (31), osteosarcoma (32), glioma (33), and non-small cell lung cancer (14). We further demonstrated that knockdown or inhibition of USP1 resulted in a reduction of Cyclin A, D, and E and an increase of CDK inhibitor p21, which are likely responsible for the growth inhibition in CRC cells.

We also examined the expression of USP1 in CRC cell lines and primary tumors by using RT-qPCR, immunoblotting, and immunohistochemistry and found that the increased level of USP1 was correlated with short overall survival of patients and with advanced stages of cancers. Interestingly, it has been shown that USP1 and its substrates participated in DNA damage response and DNA repair (6–8), whereas USP1 deficient mice were highly sensitive to DNA damage (10). Furthermore, blocking USP1 inhibited DNA repair and induced apoptosis in multiple myeloma cells (13) and sensitized lung cancer cells to cisplatin (14, 15). These data support the notion that elevated level of USP1 is required for supporting tumor cell growth and for their resistance to radio- and chemotherapies (11, 12, 34). We further tested the idea in the present study and found that knockdown or inhibition of USP1 decreased cellular anti-apoptosis proteins and enhanced the cytotoxic action of DNA-damaging agents but to a significantly less extent when 5-FU was used. Thus, highly expressed USP1 is a promising target for CRC chemotherapies, especially when combined with therapeutics that directly act on cellular DNA.

In summary, this study indicates that highly expressed USP1 is a promising target for CRC chemotherapies, especially when combined with therapeutics that directly act on cellular DNA, and provides the therapeutic target for CRC in the future.

## Data Availability Statement

All datasets generated for this study are included in the article/supplementary material.

## Ethics Statement

The studies involving human participants were reviewed and approved by The ethics committee of Xinhua Hospital, Shanghai Jiaotong University School of Medicine. The patients/participants provided their written informed consent to participate in this study.

## Author Contributions

XX, JX, and YY participated in the conception and design of the study. XX, SL, XC, KH, JW, XH, and LC performed the experiments. XX, SH, and YY interpreted the data produced, and edited the drafts of the manuscript. All authors read and approved the final manuscript.

### Conflict of Interest

The authors declare that the research was conducted in the absence of any commercial or financial relationships that could be construed as a potential conflict of interest.
